# Antioxidant, Anti-Lung Cancer, and Anti-Bacterial Activities of *Toxicodendron vernicifluum*

**DOI:** 10.3390/biom9040127

**Published:** 2019-03-29

**Authors:** Kandasamy Saravanakumar, Ramachandran Chelliah, Xiaowen Hu, Deog-Hwan Oh, Kandasamy Kathiresan, Myeong-Hyeon Wang

**Affiliations:** 1 Department of Medical Biotechnology, College of Biomedical Sciences, Kangwon National University, Chuncheon, Gangwon 24341, Korea; saravana732@gmail.com (K.S.); huxiaowen9520@gmail.com (X.H.); 2 Department of Food Science and Biotechnology College of Biotechnology and Bioscience, Kangwon National University, Chuncheon 200-701, Korea; ramachandran865@gmail.com (R.C.); deoghwa@kangwon.ac.kr (D.-H.O.); 3 Centre of Advanced Study in Marine Biology, Faculty of Marine Sciences, Annamalai University, Parangipettai 608 502, Tamil Nadu, India; kathirsum@rediffmail.com

**Keywords:** *Toxicodendron vernicifluum*, antibacterial, anti-lung cancer, MRSA NBTI, survivin

## Abstract

This work tested antioxidant, anti-lung cancer, and antibacterial activities by in vitro, in vivo, and computational experiments for the metabolites extracted from the bark, seed, and stem of *Toxicodendron vernicifluum*. The results showed that all the extracts significantly scavenged 1,2-diphenyl-1-picrylhydrazyl (DPPH) in a dose-dependent manner. But, the total phenol content (TPC) ranged from 2.12 to 89.25% and total flavonoids content (TFC) ranged from 1.02 to 15.62% in the extracts. The methanolic bark extract (MBE) exhibited higher DPPH scavenging activity than the other extracts, probably due to the higher content of the TPC and TFC present in it. Among the extracts, only the MBE showed anti-lung cancer activity at an acceptable level with a therapeutic index value (22.26) against human lung carcinoma. This was due to the cancer cell death in A549 induced by MBE through reactive oxygen species (ROS) generation, apoptosis, and cell arrest in G1 phase and inhibition of anti-pro-apoptotic protein survivin. Among the extracts, MBE showed significantly higher antibacterial activity as evident through the higher zone of inhibition 13 ± 0.5 mm against methycilin resistant strain of *Staphylococcus aureus* (MRSA), *Salmonila enteria subp. enterica*, and *P. aeruginosa*, 11 ± 0.3 mm against *E. coli* and 10 ± 0.2 mm against *B. cereus*. The MBE also showed an excellent antibacterial activity with lower minimal inhibitory concentration (MIC). Particularly, the MBE showed more significant antibacterial activity in MRSA. The in vivo antibacterial activity of the MBE was further tested in *C. elegans* model. The treatment of the MRSA induced cell disruption, damage and increased mortality of *C. elegans* as compared to the untreated and MBE treated *C. elegans* with normal OP50 diet. Moreover, the MBE treatment enhanced the survival of the MRSA infected *C. elegans*. The compounds, such as 2,3,3-trimethyl-Octane and benzoic from the MBE, metabolized the novel bacterial topoisomerases inhibitor (NBTI) and MRSA related protein (PBP2a). Overall the *T. vernicifluum* is potentially bioactive as evident by antioxidant, anti-lung cancer, and antibacterial assays. Further studies were targeted on the purification of the novel compounds for the clinical evaluation.

## 1. Introduction

The global health challenge is the burgeoning of multi-drug resistance cancer and bacterial cells. Enormous efforts are dedicated to the development of potent drug and antioxidants from natural resources against drug-resistant cancer and pathogens [1,2,3,4,5]. However, several issues do exist in the discovery of the novel drugs, such as toxicity, drug selectivity, and smart sensing of target. Plant derived metabolites are used as traditional medicine to cure several diseases because of their remarkable properties, such as anti-inflammatory, antimicrobial, antidiabetics, antioxidants, anticarcinogenic, antidepression, antiobesity, immunostimulatory, and antianxiety [6,7,8,9]. These health beneficial properties of plant-derived metabolites are due to the existence of novel flavonoids, simple phenolics, anthocyanins, and phenolic acids [7,10]. Thus, current modern chemotherapy research seeks new metabolites from plant resources.

In general, food nutrients are converted into energy through oxidative degradation by digestive enzymes. In fact, during the energy conversion, food nutrients are not only sensory counter-productive but also produce harmful pro-oxidant, such as peroxides. Pro-oxidants damage the tissues, cause DNA mutation and trigger oxidative stress through reactive oxygen species (ROS) and free radicals, which result in several chronic diseases, including gastric ulcers, atherosclerosis, acne, and cancer [11]. Thus, the appropriate balancing of the antioxidants is essential to maintain or remove the pro-oxidants or free radicals and regulate the oxidation and auto-oxidation process [11]. Traditional knowledge gained attention with natural antioxidants, such as tocopherols, flavonoids, phenols, from plants as medicine for oxidative stress-related diseases [12]. Further, plant extracts are known to inhibit the human bacterial pathogens, cancer cells, and inflammations [11,13,14]. Moreover, chronic inflammations can lead to several diseases including periodontitis, ischemic heart disease, cancers, rheumatoid arthritis, asthma, atherosclerosis, and ulcerative colitis [15,16].

Globally cancer is a dangerous disease which frequently increased morbidity and mortality. Thus, the discovery of novel metabolites against cancer cells is of a interest in current biomedicine. According to the American Cancer Society estimates 2019 report, human lung cancer occurs in about 228,150 cases causing 142,670 mortalities in USA [17]. Hence, the utilization of chemotherapeutic agents from folk medicine to target apoptosis is worth finding. The novel anticancer agents were analysed by a combination of wet laboratory assay and computational techniques. Depending on this hypothesis, targeting the apoptosis-related protein is a hallmark for the development of chemotherapeutic agents. The inhibitor of the apoptosis protein (IAPs) family is regulatory stimulus against the apoptosis in malignancies of cancer cells [18]. Survivin (BIRC5-baculoviral IAP repeats containing 5) is a member of IAPs family, significantly associated with the regulation and survival of tumor cells [19]. Survivin selectively occurs in the embryonic tissue and tumor cells and not in normal cells, and, hence, it is an important prognostic marker of apoptosis in cancer cells [20]. Moreover, survivin can inhibit the expression of the apoptosis-related protein, such as caspase 3 and caspase 9 [21]. Thus, targeting the survivin protein can result in the activation of apoptosis-related protein expression in intrinsic and extrinsic pathways, phosphatidylinositol 3-kinase (P13K)/Akt pathway, which result in enhanced apoptosis in various cancer cells including prostate, myeloid leukemia, lung, and breast cancer [22].

The growth, virulence and toxicity, and resistance of bacterial pathogens towards the drug are dependent on various protein and gene expressions. Several studies have demonstrated that the discovery of antibacterial agents by targeting the bacterial growth-related protein can result in high virulent drugs to kill the bacterial cells. The topoisomerases II, IV, and DNA gyrase are clinically validated targets for the development of the novel antibiotics. DNA gyrase is involved in the manipulation of the topological state of the bacterial DNA, which is also essential for the survival of the bacteria [23]. New DNA gyrase inhibitors called novel bacterial topoisomerases inhibitors (NBTIs) is a potential target to inhibit the DNA gyrase expression [24]. There are several research findings which indicate the application of the NBTIs on the development of novel antibacterial agents [25,26,27]. It is also reported that targeting the NBTIs is an auspicious approach for the development of resistant strain of *Staphylococcus aureus* (MRSA) [28]. Moreover, the protein penicillin-binding protein 2a (PBP2a) in MRSA enhanced resistance on β-lactams or other antibiotics. Thus, targeting of these two proteins, NBTIs and PBP2a, can provide promising antibacterial agents against MRSA.

The East Asian indigenous tree *Toxicodendron vernicifluum*, formally known as *Rhus verniciflua* Stokes of the botanical family *Anacardiaceae* [16], has been used as folk medicine for various diseases including hepatic disorder, blood disorder, gastric problems, and infectious diseases. In addition, some of the animal experimental studies revealed the efficiency of the plant species in the treatment of liver diseases and diabetics [16]. Biomedical research studies indicate that *T. vernicifluum* extracts are bioactive and exhibit significant anti-inflammatory, antibacterial, antioxidant, neuroprotective, anticancer (gastric, hepatic, colorectal, pulmonary, pancreatic, and renal) and α-glucosidase inhibitory activities [29,30,31,32]. Furthermore, several bioactive metabolites are reported from *T. vernicifluum* extracts, which include butein, quercertin urushiol, and fisetin [16]. Hence, the present study investigated the antioxidant activity of the plant extract by 1,2-diphenyl-1-picrylhydrazyl (DPPH) assay, followed by anti-lung cancer activity by cell cytotoxicity, microscopic cell imagining assay, flow cytometer-based cell arrest assay and computational assay. In addition, this work also elucidated the in vitro, in vivo, and in silico MRSA activity of the plant extract.

## 2. Materials and Methods 

### 2.1. Chemical Compounds, Bacteria, and Animals

Propodium iodine (PI), 2’,7’-Dichlorofluorescin diacetate (DCFH-DA), ascorbic acids, dimethyl sulfoxide (DMSO), (DPPH), vitamin E (α-tocopherol), ethidium bromide (EB), acridine orange hemi salt (AO), were purchased from Sigma Aldrich, (Yongin, Korea). The cytotoxicity assay kit (EZ-CyTox- water-soluble tetrazolium) was purchased from Daeil Lab Service (Gwangiu, Korea). Fetal bovine serum (FBS), dulbecco’s modified eagle medium (DMEM), roswell park memorial institute medium (RPMI) penicillin and streptomycin were purchased from Thermo Fishers Scientific (Seoul, Korea). Mueller Hinton Broth was purchased from MB cell (Seoul, Korea). The plant *T. vernicifluum* was obtained from Wonju-Malgeun-Chamott, Wonju, Kangwon, and Republic of Korea. The bacterial pathogens including *Salmonila enteria subp.enterica* (ATCC 14028), *Pseudomonas aeruginosa* (ATCC 27853), *E. coli* (ATCC 35150), *Staphylococus aureus* (ATCC 43300), *Bacillus cereus* (KNIH28), and *Aspergillus oryzae* were obtained from Korean Culture Center of Microorganisms (KCCM) or Korean Collection for Type Cultures (Republic of Korea) and maintained in nutrient agar at 4 °C. The human lung cancer cells (A549) and embryo murine fibroblast NIH3T3 cells were received from Korea cell line Bank (KCLB) (Seoul, Korea). The roundwarm (*Caenorhabditis elegans* strain N2) was received from the Caenorhabditis Genetic Center (CGC), Minnesota, USA.

### 2.2. Preparation of the Extracts

The contaminates and allergic compounds, such as urushiol, in air-dried, powdered (720 g) bark and stem of *T. vernicifluum* (TV) were boiled 120 °C for one hour then fermented with *Aspergillus oryzae* for 35 h at 30 °C [5]. The fermented bark and stem samples were extracted three times with 2 L of ethanol for 24 h reflex using magnetic stirrer. The extracts were filtered, evaporated in reduced pressure. The combined TV extracts (TVEs) were fractionated using methanol, and water. The TVEs were named as methanolic bark extract (MBE), methanolic stem extract (MSTE), methanolic seed extract (MSE), water bark extract (WBE), water stem extract (WSTE), water seed extract (WSE), and all the extracts were concentrated using the rotary evaporator at 50 °C. The yield of the concentrated samples was calculated and preserved at 4 °C for subsequent assays.

### 2.3. Gas Chromatography–Mass Spectrometry Analysis

The volatile compounds present in TVEs (MBE and MSE) was determined using the gas chromatography–mass spectrometry (GC-MS) assay followed by matching the data with mass electronic library W8N05ST.L. The GC-MS assay was performed according to the experimental condition described elsewhere [33,34]. In brief, for the identification of the volatile compounds in the TVE, we used the GC/MS apparatus (HP Agilent Technology, 7890A California, USA), GC connected with mass detector 5973 network selective mass detector. The GC equipped with DB-5MS column (30 × 0.25 mm, 0.25 μm) and an MS ion energy El, 70 eV, temperature 280 °C and scan range 50–500 m/z. The 1 μL of sample (1/10 *v/v* in methanol) was injected in the split mode, column flow rate was 1 mL·min, helium gas was used as carrier gas, the interface temperature was 280 °C, inlet temperature was 250 °C. The interface temperature was 290 °C. The oven temperature was initiated at 40 °C for 1 min and then raised to 250 °C at a rate of 3 °C/min. The volatile compounds were predicted by matching retention indices mass electronic library W8N05ST.L.

### 2.4. DPPH Radical Scavenging Assay

Free (DPPH) radical scavenging was determined according to the protocol described elsewhere [35,36] with minor modifications. Different concentrations of TVEs (1–50 μg·mL^−1^) were mixed with 2.4 mL of DPPH (100 mM) in methanol. The reaction solution was thoroughly mixed and incubated for 30 min at room temperature in dark conditions. Control samples were prepared for comparison. The scavenging of the DPPH radical was measured at 515 nm using UV spectrophotometer (Optizen 2120UV, Daejeon, Korea). Finally, the percentage of the DPPH scavenging was calculated according to the formula described earlier [3].

### 2.5. Cytotoxicity and Cell Viability Assay

Cytotoxicity and cell viability of the TVEs were determined in the NIH3T3 and A549 cells, respectively, using water-soluble tetrazolium (WST) assay kit according to the manufacture’s instructions. In brief, at first the NIH3T3 and A549 cells were cultured in the DMEM and RPMI medium incorporated with PS in humidified 5% CO_2_ incubator at 37 °C for 24 h. After the incubation period the quality of cells, such as confluences and morphology, was determined by light microscopy, then the cells were used for the cytotoxicity and cell viability assay. For the assay, the NIH3T3 (5 × 10^4^ cells) and A549 (1 × 10^5^ cells) were seeded in the 96 well plates containing DMEM medium and RPMI medium, respectively, then incubated at the above-mentioned incubation condition until it reached the 80 to 90% confluences. After reaching the required confluences, the cells were treated with TVE for 24 h by replacing the medium with DMEM or RPMI medium with the different concentrations of TVE (0–1000 μg·mL^-1^) in the above-mentioned incubation condition. After the treatment, 10 μL of EZ-CyTox reagent was added to each well, then the absorbance was measured at 450 nm. Based on the absorbance (OD) the cytotoxicity for NIH3T3 and cell viability for A549 were calculated according to the formula described elsewhere [37]. Further, for the calculation of the therapeutic index the cytotoxicity concentration (CC50) in NIH3T3 and inhibitory concentration (IC50) in A549 cells were calculated then the therapeutic index was calculated by CC50/IC50 [38]. Among the TVEs, the MBE showed the acceptable range of the therapeutic index. Thus, MBE was selected for the further light and fluorescent microscopic and cell cycle assays.

### 2.6. Reactive Oxygen Species

The ROS generation was determined using the stain DCFH-DA [39,40]. In brief, after the treatment with different IC (25, 50, and 75) and CC (25, 50, and 75) of MBE in NIH3T3 and A549 cells, the cells were washed with PBS and incubated with a final concentration of 10 μM of DCFH-DA for 30 min at 37 °C. After incubation, the cells were washed with PBS twice, then the fluorescent intensity was measured using the fluorescent spectrophotometer (Thermo Scientific, Waltham, Massachusetts, USA). The cells were also observed under the fluorescent microscopy (Olympus CKX53, Tokyo, Japan) with an excitation wavelength of 488 nm and an emission wavelength of 525 nm. The relative ROS level was calculated by comparing the treated and the non-treated cells (100%).

### 2.7. Acridine Orange (AO)/Ethidium Bromide (EB) Staining Assay

The level of cell death due to the treatment of the MBE was determined using the acridine orange and propidium iodide (AO/EB) staining assay [40,41]. In detail, different IC and CC of MBE were treated with A549 and NIH3T3 cells, respectively, for 24 h in a humidified incubator (5% CO_2_, 37 °C). After the treatment, the cells were washed with cold PBS and stained with 1:1 ratio of AO: EB, and then observed under a fluorescent microscope with a magnification of 20x (Olympus CKX53, Tokyo, Japan).

### 2.8. Cell Cycle Assay

Cell cycle arrest in A549 cells caused by MBE was determined using PI staining and FACScaliber flow cytometer [42]. In brief, the A549 cells were treated with different IC of MBE in a humidified 5% CO_2_ incubator at 37 °C for 48 h. After treatment, the cells were collected by using a sterile cell scraper and washed with cold 3 mL of PBS then centrifuged at 2000 rpm for 3 min and then the cells were fixed in 0.5 mL of ice-cold 70% ethanol for 10 min. After fixation, the cells were collected by centrifugation at 2000 rpm for 3 min followed by washing again with 0.5 mL of PBS, then centrifuged at 2000 rpm for 3 min. Finally, the cells were counted by hemocytometer, stained with 0.5 mL PI stock (50 μg·mL^−1^ of PI and 100 μg·mL^−1^ RNase A in PBS) for 30 min and then analyzed using FACS caliber (BD FACScalibur, BD, California, USA).

### 2.9. In Vitro Antibacterial Assay

Antibacterial activity of the TVEs was tested against various bacterial pathogens by the disc diffusion method [43,44]. In detail, the fresh bacterial strains were cultured in the Müller–Hinton agar (MHA) for 24 h at 37 °C. After incubation, the pure single colonies were picked and inoculated in newly prepared Müller–Hinton broth (MHB) and incubated at 37 °C in a rotary shaker incubator at 180 rpm for 24 h. One hundred microliters of bacterial cells (109 CFU·mL^−1^) was spread on the MHA using a glass spreader. Then sterile 6 mm discs containing different concentrations (25–200 μg·mL^−1^) of TVEs were placed on the MHA plates with microbial inoculation. These plates were incubated in an incubator at 37 °C for 24 h. After the incubation period, the zone of inhibition was measured using a ruler. The experiments were determined three times, and the zone of inhibition (mm) is expressed as mean ± standard error (SEM, *n* = 3). For the determination of the minimal incubatory concentration (MIC), microdilution assay was performed according to the method described previously [45]. Further, these MIC of TVEs were subjected to growth curve determination [45].

### 2.10. In Vivo Antibacterial Assay

The in vivo anti-MRSA assay was performed based on the life span assay [46] using roundworm, grown in 35 mm diameter plates containing the Nematode growing medium (NGM) with a normal diet of 50 μL of the OP50 (*E. coli*) at 20 °C. In this assay, four treatments were applied: (i) 50 μL of OP50 (108 CFU·mL^−1^); (ii) 50 μL of MBE (120 μg·mL^−1^) +OP50 (108 CFU·mL^−1^); (iii) 50 μL of MRSA (108 CFU·mL^−1^) + 50 μL OP50 (108 CFU·mL^−1^); (iv) 50 μL of MBE (120 μg·mL^−1^) +50 μL of MRSA (108 CFU·mL^−1^) +OP50 (108 CFU·mL^−1^). For each treatment, 90 roundworms were used (30 worms/plates), then every day the mortality of the worms was counted, and the survival of worms (%) was calculated using the standard method of comparing worms counts on the first day of assay. The worms, dead/alive, under different treatments were observed under optical and fluorescent microscope stained with propidium iodide or Syto-9 (Olympus CKX53, Tokyo, Japan).

### 2.11. Computational Screening of the Compounds

A computational approach was applied to screen and predict the underlying mechanism of the potent activity of the MBE based on the GC-MS data. For the in silico antibacterial activity two important bacterial protein targets, PBP2a protein from MRSA (PDB ID:3ZFZ) [47] and novel bacterial topoisomerases inhibitors (PDB ID:4PLB) [24], and for the anti-lung cancer activity, anti-pro-apoptosis protein survivin (PDB ID: 1F3H) [21], were retrieved from the RCSB protein data bank (PDB) (https://www.rcsb.org/). Next, the structure of the compounds from the MBE detected by GCMS was generated based on canonical SMILES (Simplified Molecular Input Line Entry System) (https://www.ncbi.nlm.nih.gov/pccompound) using the ACD/ChemSketch. The pre-treatments of ligands and receptor including energy minimization and removal of the water molecules were done according to the methods described earlier [4,21,47,48]. The interactions between the MBE compounds (ligands) and targeted protein (receptor) and their molecular docking score (Kcal·mol^−1^) were analyzed by using the Argus Lab 4.0.1 (Mark Thompson and Planaria Software LLC). These interactions and distance were also observed using the BIOVIA Discovery Studio 2016 (Accelrys Software Inc., San Diego, CA, USA).

## 3. Results and Discussion

*T. vernicifluum* is used as folk medicine to cure various diseases, such as inflammatory diseases, abdominal disorders, and cancers in the Republic of Korea [49]. Although this plant species has received attention in herbal therapy, its utilization is limited due to its toxic substance, urushiol, that can cause inflammation and irritation to people who are sensitive [50]. The toxicant urushiol is an allergic substance present in the plant family of *Anacardiaceae* [5]. Moreover, these toxicants are removed by using the several pretreatment methods including the heat treatments, far-infrared radiation, enzyme treatments and solvent extracts [31,51]. The present study removed the toxicant by heat treatments and fungal fermentation [5], and the absence of the urushiol was also confirmed by high performance liquid chromatography (HPLC). Further, the TVEs were tested by cytotoxicity assay with NIH3T3 cells followed by antioxidant, antibacterial, and anti-lung cancer. The yields of TVEs were 18.2, 2.4, 0.6, 0.63, 0.62, and 0.08 for WBE, WSTE, WSE, MBE, MSTE, and MSE, respectively. Although antioxidant, anticancer, and antibacterial activities of the TVEs are known from earlier works, there is no study to elucidate anti-lung cancer and anti-MRSA activities of TVEs. Hence, this study used both wet laboratory and bioinformatics computational experiments followed by in vivo anti-MRSA using the roundworm (*C. elegans*) for screening the novel molecules of the plant species for future pharmacological applications.

### 3.1. Gas Chromatography–Mass Spectrometry (GC-MS) Based Identification Metabolites

The bioactive extracts of MBE and MSTE were subjected to GC-MS analysis to identify the compounds, and the results are presented in Table 1. Through matching the chromatography data with electronic mass library W8N05SR.L, the molecular mass, chemical abstract service (CAS) number and international union of pure and applied chemistry (IUPAC) names of the TVEs compounds were detected. According to the GC-MS results, the MBE contained compounds, such as hexamethylcyclotrisiloxane, octamethylcyclotetrasiloxane, 2,3,3-trimethyl-Octane, 2,5-Bis-(4-bromophenyl)pyridine, 1-Ethenyl-4-methylbenzene, benzoic acid, Benzene, 1-ethoxy-2-(2-nitro-1-propenyl)-,(E)-(9CI), and Pentadioic acid, dihydrazide N2,N2’-bis(2-furfurylideno), while the MSTE contained pyrazolo [1,5-a]pyridine, N,N-dimethylquinolin-2-amine, Benzaldehyde, Methyl-bis(trimethylsilyloxy)silicon, and Tetrasiloxane. These compounds are already known to have antioxidant, antimicrobial, cytotoxicity, anticancer, and anti-inflammatory activities [52,53,54].

### 3.2. Antioxidant Assay

**Figure 1:**
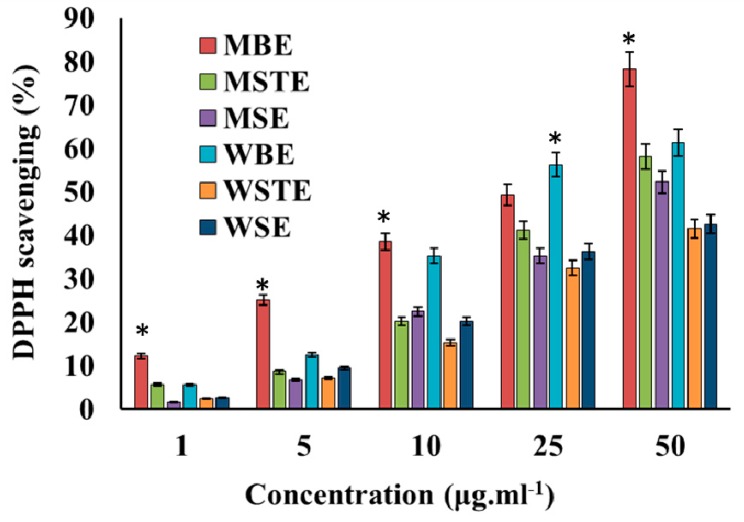
Antioxidant activity; 1,2-diphenyl-1-picrylhydrazyl (DPPH) scavenging efficiency of the methanolic and water extracts of *T. vernicifluum*. Methanolic seed extract (MSE), methanolic bark extract (MBE), methanolic seed extract (MSTE), water seed extract (WSE), water bark extract (WBE), water stem extract (WSTE). Data are mean ± standard error (SEM, *n* = 3). * *p* < 0.05 significantly differs with other extracts.

Antioxidant activity of the TVEs was determined in a DPPH scavenging assay. All the extracts significantly scavenged DPPH in a dose dependent manner (Figure 1). This activity also significantly varied between different types of TVE. Among the extracts, MBE exhibited higher activity (Figure 1). The total phenol content (TPC) and flavonoids content (TFC) were determined in the TVEs. The TPC ranged from 2.12 to 89.25%, and TFC varied from 1.02 to 15.62% in TVEs and both were found higher in the MBEs. Probably the higher content of the TPC and TFC present in the MBEs might be responsible for the higher antioxidant DPPH scavenging activity than other TVEs as there is a possible correlation between TPC and antioxidant activities [55,56,57]. The TVE is reported to contain bioactive phytochemicals including polyphenols, and flavonoids [5,57,58,59]. The TV has hydroxyl radicals [60] that protect human low-density lipoprotein (LDH), scavenge DPPH and –OH radicals and inhibit the linoleic acid oxidation [61,62]. The fruit extract of TV has glucose, glucose oxidase, and scavenge hydroxyl and free radicals [63].

### 3.3. Anti-Lung Cancer Activity

**Figure 2:**
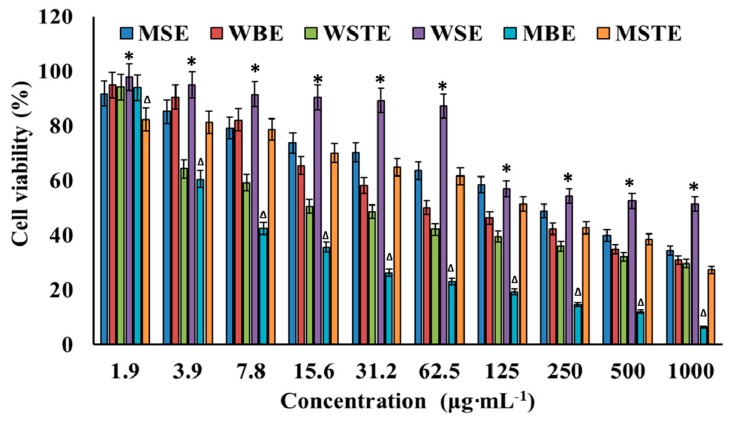
Cytotoxicity of different methanolic and water extracts of *T. vernicifluum*. Methanolic seed extract (MSE), methanolic bark extract (MBE), methanolic seed extract (MSTE), water seed extract (WSE), water bark extract (WBE), water stem extract (WSTE). Data are mean ± standard error (SEM, *n* = 3). * *p* < 0.05 is significantly non-cytotoxic to A549 cells than other types of extracts. ∆*p* < 0.05 is significantly cytotoxic to A549 cells than other types of extracts.

In this experiment, cytotoxicity of TVEs was tested against normal cell line NIH3T3 followed by the cell viability in human lung carcinoma cells A549. TVEs decreased the cell viability (Figure 2) and increased the cytotoxicity (Figure S1) with increasing concentrations in a dose-dependent manner. The natural compounds could be considered to be worthy of further evaluation if it achieved the therapeutic index ≥16 [64]. Thus, the determination of the therapeutic index is essential for safe clinical therapy [65,66]. In the present study, among TVEs, only the MBE was found to be at an acceptable level with a therapeutic index value of 22.26 against human lung carcinoma (Table 2). The value is greater than 16, and, hence, the MBE was used further for anticancer study. The cytotoxicity study in normal cell (NIH3T3) experiments, in addition to the primary WST assay by light microscopy and fluorescent microscopy assay indicated that the MBE induced the cell damage, ROS generation and apoptosis cells with the increase of the CC in a dose-dependent manner (Figure S2a,b).

**Figure 3:**
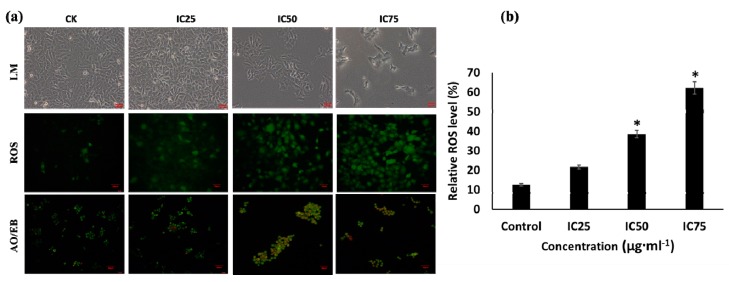
(**a**) Effect of the methanolic and water extracts of *T. vernicifluum* on cellular changes in A549 cells. (**b**) Relative reactive oxygen species level. IC—inhibitory concentration, LM—light microscopic images, ROS—reactive oxygen species, AO/EB—acridine orange and propidium iodide staining. Data are mean ± standard error (SEM, *n* = 3). The level of ROS is * *p* < 0.05 significantly varied with control treatment. The red scale bar in micrographs indicated the length of scale is 50 µm.

**Figure 4:**
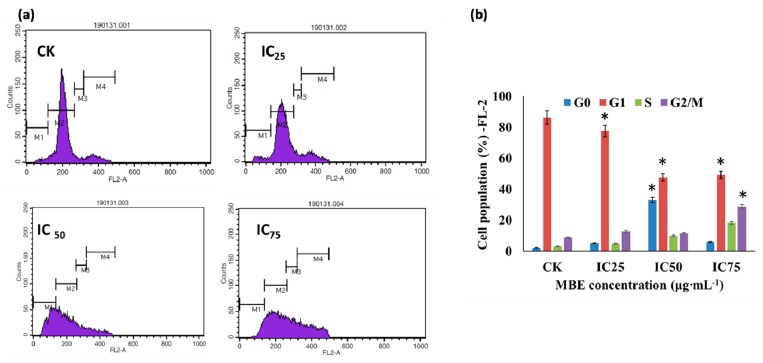
(**a**) Arrested the cell cycle of the human lung carcinoma A549 cells by the MBE treatments. Distribution of the cell cycle as determined by propodium iodide staining after the treatment of various concentrations of MBE. (**b**) Proportion of cells per cell phase in cell cycle. Data are as mean ± standard error (SEM, *n* = 3). * *p* < 0.05; M1—G0; M2—G1; M3—S and M4—G2/M.

The drugs destroy multiplication of cancerous cells through various mechanisms including intrinsic or extrinsic pathway in cancer cell death, apoptosis, ROS generation, and cell cycle arrest [66,67,68,69]. The apoptosis occurs by the enzymatic cleavage of oligonucleosomal sections, affecting the plasma layer phospholipids asymmetry, and disturbing the cell division [70]. In the present study, the treatment of IC75 of MBE showed higher cytotoxicity, ROS generation, and apoptotic cells as compared to the cells treated with IC50, IC25 and untreated control cells (Figure 3a). Particularly the ROS level significantly varied between the treatments, and it was found higher in IC75 of MBE treatment (Figure 3b). To elucidate the mechanism of MBE induced anti-lung cancer activity, the cell cycle distribution was determined using the FACS caliber using PI staining. After the treatment of different IC of MBE, the distribution of the cells in the cell cycle stage varied, as presented in Figure 4. The cell population in growing cells (G1 phase) was significantly high in the untreated group as compared to MBE treated groups. As indicated in the G1 phase healthy cells were found in the MBE treatment and untreated control. The treatments at IC50 and IC75 significantly increased apoptosis cells (G0 phase) (Figure 4b). This result indicated that MBE proliferation of the A549 cells was arrested in G1 phase and this is in accordance with previous work [71]. Perhaps the cyclin complex D/CDK4 may play a vital role in the regulation of the cell cycle phase and cyclin E/CDK2 and A/CDK2 complexes are associated with the S phase [72,73]. Thus, the present result of G1 phase was arrested by the down-regulation of the cylclin A, D, E, CDK2 and CDK4 by MBE treatment.

**Figure 5:**
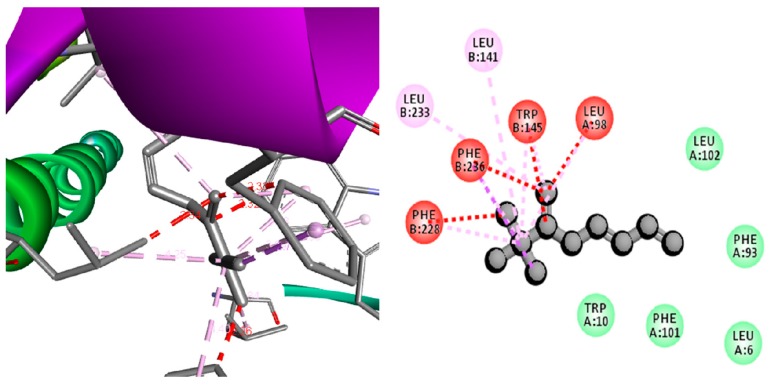
3D and 2D docking pose showing the interactions between compounds 2,3,3-trimethyl-octane and anti-pro apoptotic protein survivin (1F3H).

Further, this study analyzed the inhibitory effect of the MBE compounds on anti-pro-apoptotic protein—survivin by computational molecular docking method. The results indicated several compounds from MBE significantly metabolized survivin, which resulted in the inhibition of survivin expression towards the increment of the pro-apoptotic protein expression that leads to cell ablation [21]. All the compounds from the MBE showed a good negative docking score (Table 1); however, among the test compounds, 2,3,3-trimethyl-Octane (−14.9 Kcal/mol), 2,5-Bis-(4-bromophenyl)pyridine (−14.01 Kcal/mol), 1-Ethenyl-4-methylbenzene (−12.87 Kcal/mol), and Benzene, 1-ethoxy-2-(2-nitro-1-propenyl)-, (E)- (9CI) (−11.52 Kcal/mol) showed a higher docking energy than the commercial anti-inflammatory drug celecoxib (−11.49 Kcal/mol). One of the compounds, namely 2,3,3-trimethyl-Octane, established a strong interaction with survivin (1F3H) by interacting with amino acids, such as hydrophobic side chain Leu 233, Leu 141, Phe 228, Phe 236, Trp 145, and Leu 98 (Figure 5). The potential anti-lung cancer activity of MBE was not only related to the 2, 3, 3-trimethyl-Octane (Figure S4) but also associated with synergism of various compounds present in MBE. Similarly, the phenolic and flavonoid substances from TV show anticancer activity against various cancer cell lines including Human B lymphoma [74], non-small lung carcinoma, ovary malignant ascites, skin malenama, colon adenocarcinoma [5], neuronal HT22, microglial BV2 cells [75], and human osteosarcoma [76]. Overall these results indicated that MBE induced lung cancer cell death through the activation of the ROS and cell arrest followed by the inhibition of anti-pro apoptotic protein survivin.

### 3.4. Antibacterial Activity

**Figure 6:**
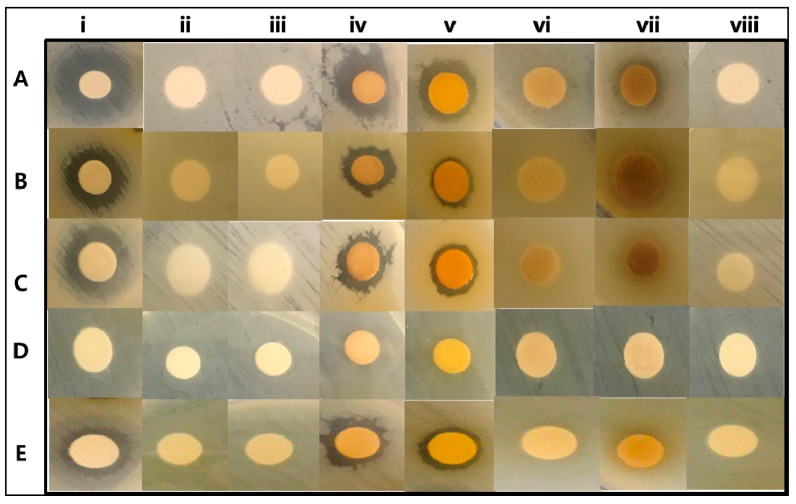
In vitro antibacterial activity of *T. vernicifluum*. *Staphylococcus aureus* (**A**), *Salmonila enteria subp.enterica* (**B**), *Pseudomonas aeruginosa* (**C**), *E. coli* (**D**), and *Bacillus cereus* (**E**); Antibiotics oxacillin (i), 200 µL of methanolic alone (ii), MSE (iii), MBE (iv), MSTE (v), WSTE (vi), WBE (vii), WSE (viii). methanolic seed extract (MSE), methanolic bark extract (MBE), methanolic seed extract (MSTE), water seed extract (WSE), water bark extract (WBE), water stem extract (WSTE).

The effect of TVEs on various bacterial pathogens was tested in vitro well diffusion and microdilution assay followed by computational targeting of bacterial growth and cell wall-related protein. The results indicated that TVEs showed antibacterial activity in dose-dependent manner (Figure S3a–e, Figure 6). Although the TVEs showed significant antibacterial activity on tested bacterial pathogens, a higher inhibition zone was recorded with *S. aureus* (MRSA). Among the TVEs, MBE showed a higher zone of inhibition 13 ± 0.5 mm against MRSA, *S. enteria subp. enterica*, and *P. aeruginosa*, 11 ± 0.3 mm against *E. coli,* and 10 ± 0.2 mm against *B. cereus* (Figure S3a–e). The present work also determined the minimal inhibitory concentration (MIC) of TVEs against various bacterial pathogens (Table 3).

**Figure 7:**
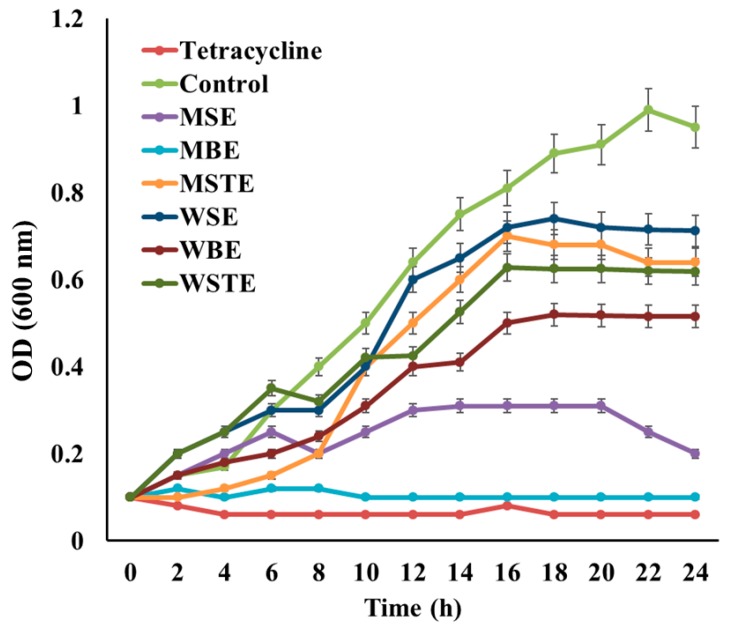
Analysis of the growth curve for antimicrobial activity of MIC concentration of different plant extracts against MRSA. methanolic seed extract (MSE-MIC:10.24 µg·mL^−1^), methanolic bark extract (MBE-MIC:7.12 µg·mL^−1^), methanolic seed extract (MSTE-MIC:9.25 µg·mL^−1^), water seed extract (WSE-MIC:8.75 µg·mL^−1^), water bark extract (WBE-MIC:9.21 µg·mL^−1^), water stem extract (WSTE-MIC:9.45 µg·mL^−1^). Data are mean ± standard error (SEM, *n* = 3). * *p* < 0.01 significantly varied with control.

**Figure 8:**
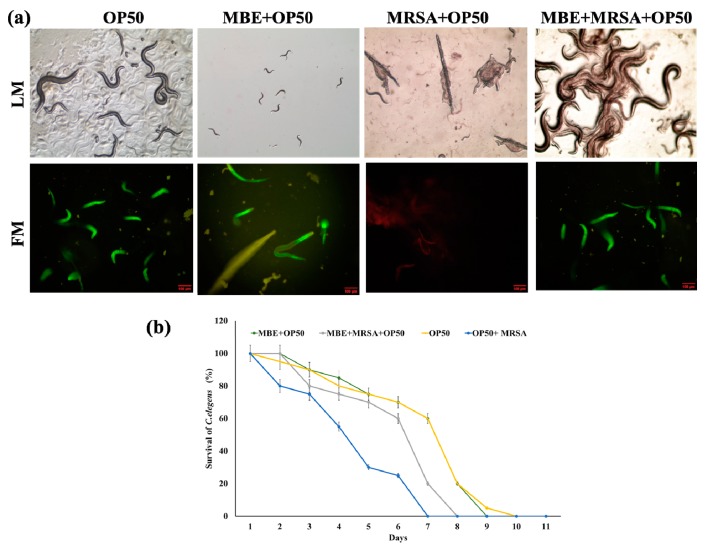
*C. elegans* life span assay. (**a**) Optical (LM) and fluorescent (FM) microscopic (10x magnification) observation of the live dead *C. elegans* after the propidium iodide or Syto-9 staining. (**b**) Determination of the *C. elegans* survival (%) during the 10 days of life span assay. Data are mean ± standard error (SEM, *n* = 3). * *p* < 0.01 significantly varied with negative control. OP50—*E. coli* OP50 normal food diet, MRSA—methicillin resistant *Staphylococcus aureus*, MBE—methanolic bark extract. The red scale bar in micrographs indicated the length of scale is 100 µm.

MIC significantly varied between the type of TVEs, and bacterial pathogens. Similarly, the extracts derived from the TV is shown to have antibacterial activity against gram-negative bacterial pathogen, *E. coli* [31]. Among various TVEs, MBEs showed excellent antibacterial activity against bacterial pathogens with lower MIC. Particularly, MBE showed significant antibacterial activity in MRSA (Figure S3f). These findings are also evident in the growth curve study (Figure 7). Hence, MBE was selected for further in vivo and computational based antibacterial assay. The in vivo antibacterial activity of the MBE was tested in *C. elegans* animal experiments. The treatment with MRSA induced the cell disruption, damage, and increased mortality of *C. elegans* as compared to the untreated and MBE treated *C. elegans* with normal OP50 (Figure 8a), which indicated the nontoxicity and acceptability of the dose for the in vivo assay in C. elegans. Moreover, the treatment of MBE increased the survival of the MRSA infected *C. elegans* (Figure 8a,b).

**Figure 9:**
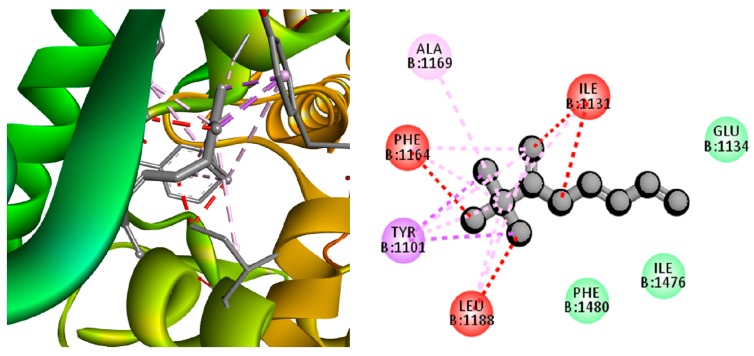
3D and 2D docking pose showing the interactions between 2,3,3-trimethyl-Octane and bacterial topoisomerase inhibitors (NBTIs) protein.

**Figure 10:**
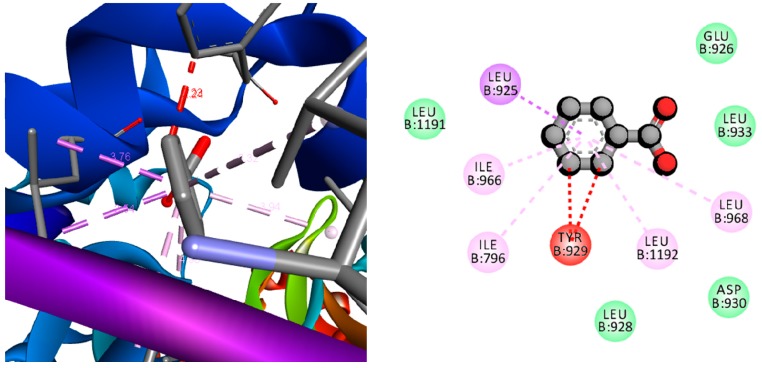
3D and 2D docking pose showing the interactions between benzoic acid and methycilin resistant strain of *Staphylococcus aureus* (MRSA) related protein.

The molecular mechanism of MBE induced bacterial cell death as investigated by computational study and socking score is presented in Table 1. The results indicated that among the test compounds from the MBE, 2,3,3-trimethyl-Octane showed a high docking score (−13.74 Kcal/mol) against NBTI (4PLB) with the strong interactions in amino acids, such as hydrophobic side chain such as Ala1169, Leu 1188, Tyr1101, Phe1164, and Ile1131, electrically charged side chain Glu1134, (Figure 9). Furthermore, another compound benzoic acid exhibited a high docking score (−12.72 Kcal/mol) with interactions in hydrophobic side chain Leu925, Leu968, Leu1192, Tyr929, Ile796, Ile966, and Leu 925 of MRSA related protein (3ZFZ) (Figure 10). The in vitro, in vivo, and computational studies indicated that the antibacterial activity of MBE was due to the synergism of various compounds present in the MBE. The growth of bacterial pathogens can be inhibited through targeting of the growth and virulence protein topoisomerases II, IV, and DNA [23] as the new DNA gyrase inhibitor NBTIs is a potential target [24]. There are several works that indicate that targeting the NBTIs can be an excellent strategy for the development of a novel antibacterial agent [25,26,27] against MRSA [28]. Moreover, the penicillin-binding protein 2a (PBP2a) in MRSA will enhance resistance on β-lactams or other antibiotics. Similarly, in the present study, the compounds from MBE significantly metabolized the targeted proteins, which probably resulted in the inhibition of this protein expression in bacterial cells in favor of bacterial cell death. In addition, this work indicated that compounds, such as 2, 3, 3-trimethyl-Octane and benzoic acid from the MBE, were potent compounds against MRSA over the commercial antibiotic oxacillin (Table 1).

## 4. Conclusions

This work extracted metabolites from bark, seed, and stem of *T. vernicifluum* in water and methanol and then tested for antioxidant, antibacterial, and anti-lung cancer activities. The methanolic bark extract (MBE) was found potent against the human lung cancer cell line and bacterial pathogens. MBE triggered human lung cancer cell death through the oxidative stress (ROS), apoptotic pathway, cell arrest (G1 phase) followed by down-regulation of survivin. In the case of antibacterial activity, MBE killed the bacterial pathogens through targeting NBTI and PBP2 as evident by computation study. In vivo antibacterial activity revealed the nontoxicity and acceptability of the MBE for the growth of *C. elegans* and survival of the MRSA infected worms. Overall, this work concluded that *T. vernicifluum* is an excellent source of bioactive substances towards developing drug leads.

## Figures and Tables

**Table 1:** Analysis of alkaloids and low molecular weight molecules from the methanolic extract of bark (MBE) and stem (MSTE) of *T. vernicifluum* by gas chromatography–mass spectrometry (GC-MS) and their molecular instructions with antibacterial activity and anti-proapoptotic related proteins. The bold values indicate a higher docking score than other compounds.

Extracts	IUPAC Name of Compound	Retention Time	Area (%)	Molecular Weight (g/mol)	Antibacterial Protein		Anti-Pro Apoptotic Protein
					4PLB	3ZFZ	1F3H
MSTE							
1	Pyrazolo[1,5-a]pyridine	21.73	2.56	118.14	−7.34	−7.51	−8.02
2	N,N-dimethylquinolin-2-amine	20.957	28.19	172.231	−8.50	−8.95	−10.61
3	Benzaldehyde	22.721	5.88	106.124	−8.41	−9.46	−10.15
4	methyl-bis(trimethylsilyloxy)silicon	23.063	4.13	221.498	−7.98	−8.98	−10.44
5	Tetrasiloxane	24.158	7.45	170.417	−3.56	−3.57	−3.51
MBE							
1	Hexamethylcyclotrisiloxane	3.455	17.59	222.46	−7.40	−8.29	−9.34
2	Octamethylcyclotetrasiloxane	5.562	1.15	296.62	−7.76	−7.92	−10.82
3	2,3,3-trimethyl-Octane	15.809	1.56	156.308	**−13.74**	−11.25	**−14.9**
4	2,5-Bis-(4-bromophenyl)pyridine	18.404	0.92	389.084	−10.12	−11.04	−14.01
5	1-Ethenyl-4-methylbenzene	20.915	4.09	117.171	−9.77	−9.01	−12.87
6	Benzoic acid	20.915	16.38	122.123	−8.39	**−12.72**	−9.88
7	Benzene, 1-ethoxy-2-(2-nitro-1-propenyl)-, (E)- (9CI)	22.721	1.63	207.226	−9.45	−9.34	−11.52
8	Pentadioic acid, dihydrazide N2,N2’-bis(2-furfurylideno)	23.022	1.13	316.312	−9.36	−9.3	−9.51
Antibiotic							
	Oxacillin	-	-	-	−11.02	−10.25	-
Cancer Drug							
	Celecoxib				-	-	−11.49

**Table 2:** Evaluation of the therapeutic index of various extracts derived from *T. vernicifluum*. MeOH seed extract (MSE), MeOH bark extract (MBE), MeOH seed extract (MSTE), water seed extract (WSE), water bark extract (WBE), water stem extract (WSTE). ND- Not detected.

Extract Code	CC50 in NIH3T3 (µg·mL^−1^)	IC50 A549 (µg·mL^−1^)	Therapeutic Index (CC50/IC50) (µg·mL^−1^)
MSE	500	218.75	2.28
MBE	130.25	5.85	22.26
MSTE	62.5	156.25	0.40
WSE	ND	1000	0
WBE	187.5	46.8	4.00
WSTE	15.6	23.4	0.66

**Table 3:** Minimal inhibitory concentration of various extracts derived from *T. vernicifluum* extracts on bacterial pathogens. MeOH seed extract (MSE), MeOH bark extract (MBE), MeOH seed extract (MSTE), water seed extract (WSE), water bark extract (WBE), water stem extract (WSTE). Data are mean ± standard error (SEM, *n* = 3).

Pathogens	Concentration (µg·mL^−1^)					
	MSE	MBE	MSTE	WSE	WBE	WSTE
*S. aureus*	10.24 ± 1.1	7.12 ± 0.8	9.25 ± 0.2	8.75 ± 0.2	9.21 ± 0.8	9.45 ± 1.5
*S. enteria subp.enterica*	15.24 ± 2.1	11.58 ± 1.2	15.24 ± 2.1	15.75 ± 1.5	20.52 ± 0.6	16.52 ± 1.5
*P. aeruginosa*	8.95 ± 0.8	8.52 ± 1.5	11.54 ± 1.9	14.52 ± 1.6	17.24 ± 0.8	14.65 ± 1.4
*E. coli*	15.2 ± 1.9	9.45 ± 1.6	9.85 ± 1.2	18.65 ± 1.8	15.45 ± 0.4	21.45 ± 1.8
*B. cereus*	8.56 ± 0.5	5.86 ± 1.4	12.15 ± 1.2	18.45 ± 1.4	10.25 ± 1.2	19.45 ± 2.1

## Supplementary Materials

The following are available online at https://www.mdpi.com/2218-273X/9/4/127/s1, Figure S1: Cytotoxicity of different methanolic and water extracts of *T. vernicifluum*. Figure S2: Effect of the methanolic and water extracts of *T. vernicifluum* on cellular changes in NIH3T3 cells. Figure S3: Zone of inhibition determined by disk diffusion Antibacterial assay against methicillin-resistant *Staphylococcus aureus*-MRSA. Figure S4: GC-MS results-based evidence of 2,3,3-trimethyl-Octane and benzoic acid in MBE of *T. vernicifluum*.
