# Supplementary material for: Antioxidant, Anti-Lung Cancer, and Anti-Bacterial Activities of Toxicodendron vernicifluum
Source: Biomolecules. 2019 Mar 29;9(4):127. doi: 10.3390/biom9040127 (PMC6523688; doi:10.3390/biom9040127)
Supplement: Supplementary file 1 [file biomolecules-09-00127-s001.pdf]

Antioxidant, anti-lung cancer and anti-bacterial activities of *Toxicodendron vernicifluum*

SUPPLEMENTARY INFORMATION

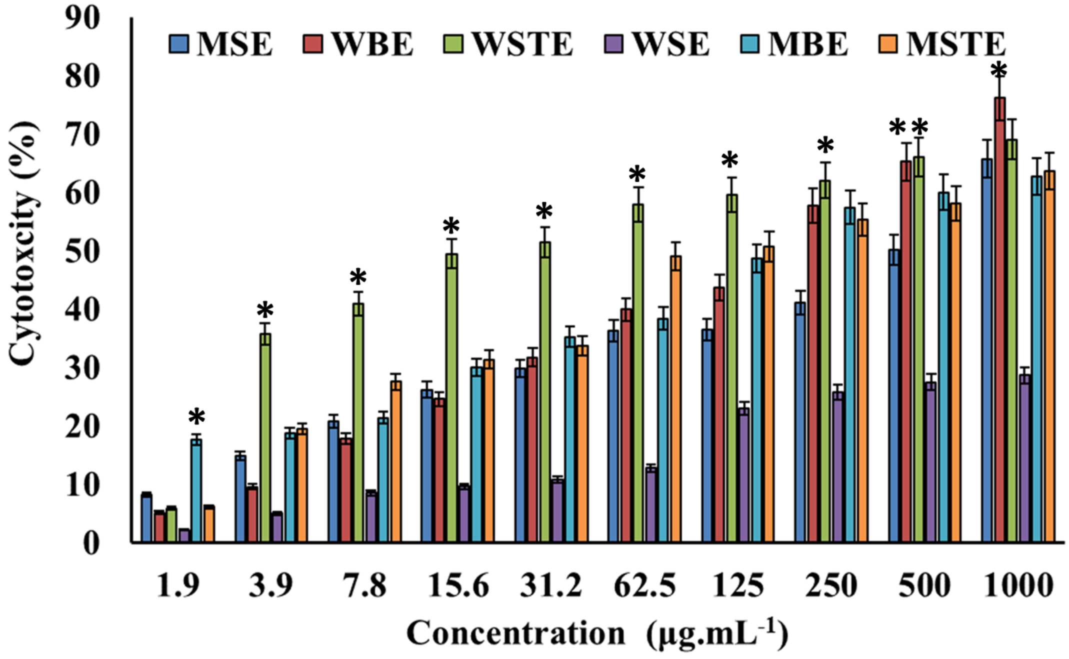

SI.Fig.1. Cytotoxicity of different methanolic and water extracts of *T. vernicifluum.* MeOH seed extract (MSE), MeOH bark extract (MBE), MeOH seed extract (MSTE), water seed extract (WSE), water bark extract (WBE), water stem extract (WSTE). **p* <0.05 is significantly cytotoxic to NIH3T3 cells than other type of extracts.

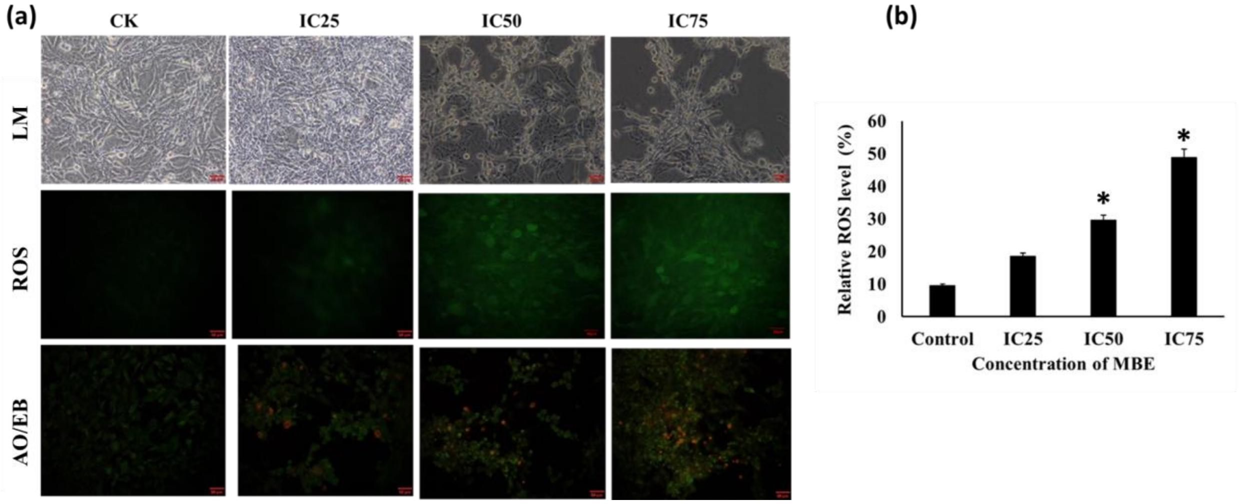

SI.Fig.2. Effect of the methanolic and water extracts of *T. vernicifluum* on cellular changes in NIH3T3 cells (a), relative reactive oxygen species level (b). IC-inhibitory concentration, LM-light microscopic images, ROS-reactive oxygen species, AO/EB- acridine orange and propidium iodide staining. The level of ROS is

*p <0.05 significantly varied with control treatment. The red scale bar in micrographs indicated the length of scale is 50 µm.

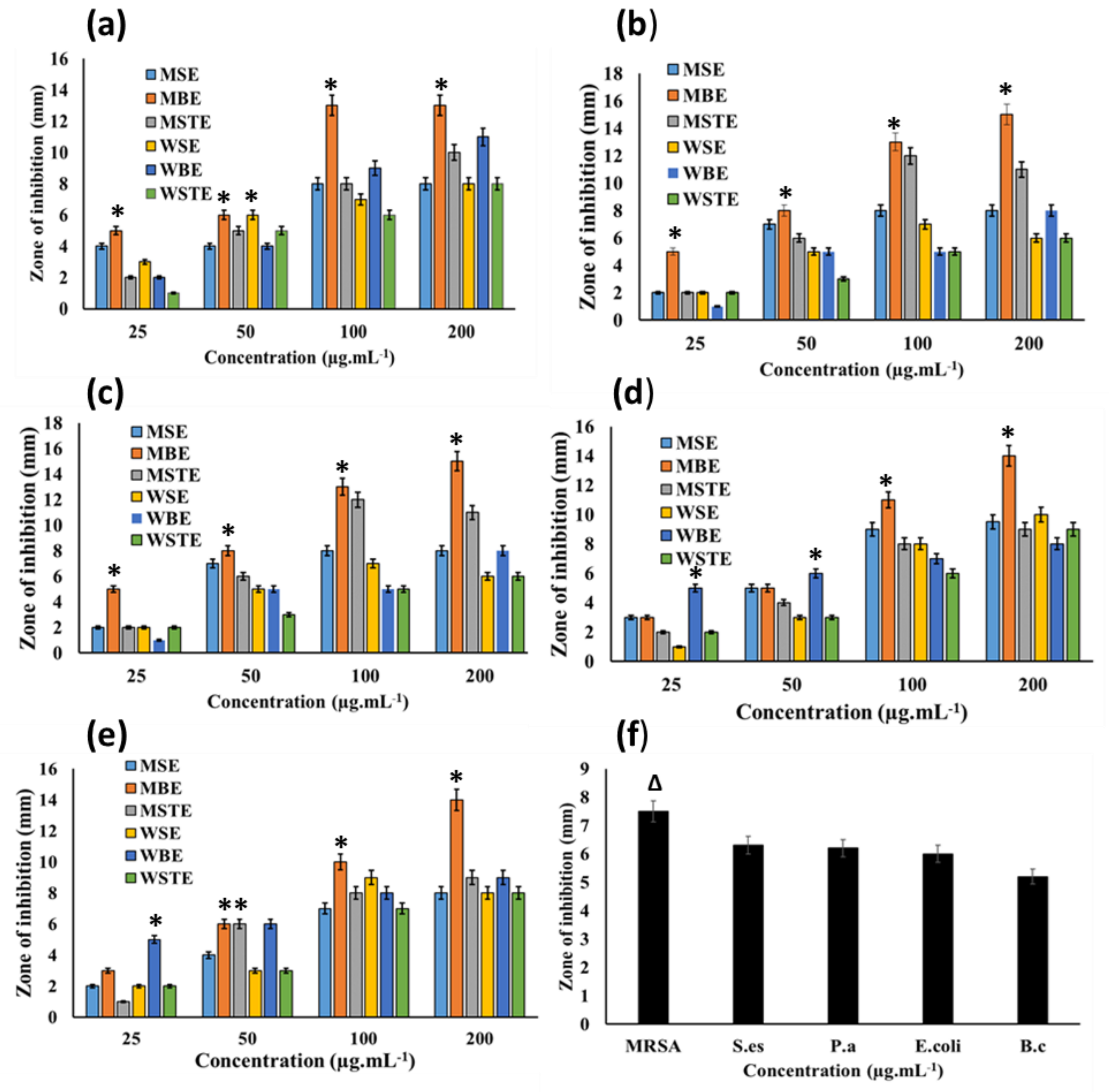

SI.Fig.3. Zone of inhibition determined by disk diffusion Antibacterial assay against methicillin-resistant *Staphylococcus aureus*-MRSA (a), *Salmonila enteria subp.enterica*-P.es (b), *Pseudomonas aeruginosa*-P.a (c), *E. coli* (d), *Bacillus cereus*- B.c (e), Cumulative inhibition rate against various pathogens (f). MeOH seed extract (MSE), MeOH bark extract (MBE), MeOH seed extract (MSTE), water seed extract (WSE), water bark extract (WBE), water stem extract (WSTE). Data are mean ± standard error (SEM, n=3). *p<0.05 significantly higher than other extracts. Δ indicated the higher inhibitory efficiency of MBE on MRSA.

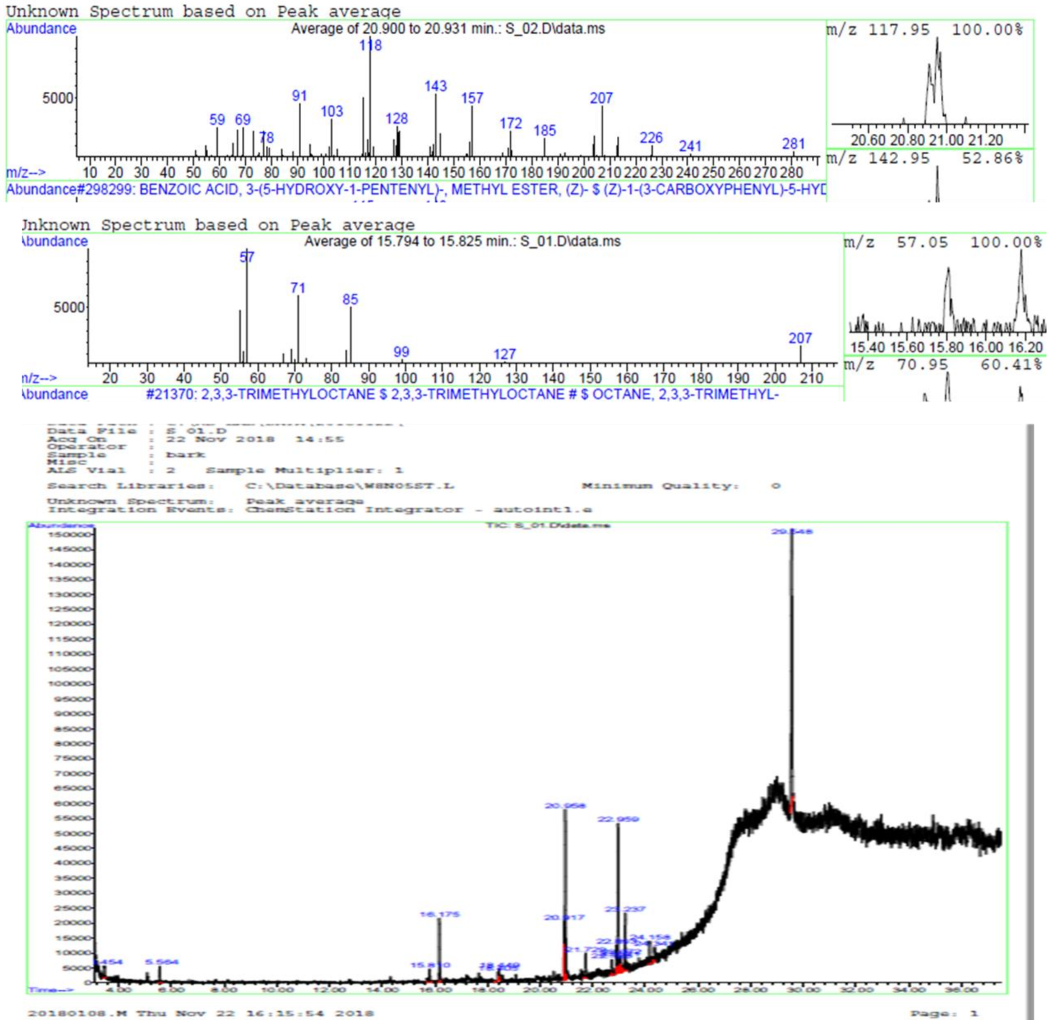

SI.Fig.4. GC-MS results based evidence of 2,3,3-trimethyl-Octane and benzoic acid in MBE of *T. vernicifluum*
